# Time-Varying Spectral Kurtosis: Generalization of Spectral Kurtosis for Local Damage Detection in Rotating Machines under Time-Varying Operating Conditions

**DOI:** 10.3390/s21113590

**Published:** 2021-05-21

**Authors:** Jacek Wodecki

**Affiliations:** Faculty of Geoengineering, Mining and Geology, Wroclaw University of Science and Technology, Na Grobli 15, 50-421 Wroclaw, Poland; jacek.wodecki@pwr.edu.pl

**Keywords:** kurtosis, spatial filtering, time-frequency analysis, vibration, local damage detection

## Abstract

Vibration-based local damage detection in rotating machines (i.e., rolling element bearings) is typically a problem of detecting low-energy cyclic impulsive modulations in the measured signal. This can be challenging as both the amplitude of a single damage-related impulse and the distance between impulses might be changing in time. From the signal processing point of view, this means time varying regarding the the signal-to-noise ratio, location of information in the frequency domain, and loss of periodicity (this remains cyclic but not periodic). One of the many attempted approaches to this problem is filtration using custom filters derived in a data-driven fashion. One of the methods to obtain such filters is a selector approach, where the value of a certain statistic is calculated for individual frequency bands of a signal that results in the magnitude response of a filter. In this approach, each chosen statistic will yield different results, and the obtained filter will be focused on different frequency bands focusing on different behaviors. One of the most popular and powerful selectors is spectral kurtosis as popularized by Antoni, which uses kurtosis as an operational statistic. Unfortunately, after closer inspection, it is easy to notice that, although selectors can significantly enhance the signal, they accumulate a great deal of noise and other background content of signals, which occupies the space (or rather time) in between the impulses. Another disadvantage is that such filters are time-invariant, because, in the principle of their construction, they are not adaptive, and even slight changes in the signal yield suboptimal results either by missing relevant data when the conditions in the signal change (i.e., informative impulses widen in bandwidth), or by accumulating unnecessary noise when the relevant information is not present (in between impulses or in frequency bands that impulses no longer occupy). To address this issue, I propose generalization of the selector approach using the example of spectral kurtosis. This assumes creating a time-varying selector that can be seen as a spatial filter in the time-frequency domain. The time-varying SK (TVSK) is estimated for segments of the signal, and, instead of a vector of SK-based filter coefficients, one obtains a TVSK-based matrix of coefficients that takes into account the time-varying properties of the signal. The obtained structure is then binarized and used as a filter. The presented method is tested using a simulated signal as well as two real-life signals measured on heavy-duty bearings in two different types of machine.

## 1. Introduction

From the analytical point of view, vibration-based local damage detection in rotating machines (i.e., rolling element bearings) is typically a problem of detecting low-energy cyclic impulsive modulations in the measured signal, often in the form of cyclic impulses. Over the years, many different techniques have been developed, such as time-frequency analysis [1,2,3,4,5], model-based analysis [6,7], amplitude and phase demodulation [8], amplitude and frequency modulation analysis [9], cepstrum analysis [10,11], empirical mode decomposition [12,13,14], and many others.

One of the many attempted approaches to this problem is filtration using custom filters derived in a data-driven fashion. They can be constructed in several different ways. The first method is a result of designing a classic digital filter, such as a finite impulse response filter, in a way that it is optimized for a particular signal [15]. The second way is an approach where the value of a certain statistic is calculated for individual frequency bands of a signal, which results in a magnitude response of the filter. In this approach, each chosen statistic will yield a different result, and the obtained filter will “select” different frequency bands depending on the chosen working statistic, focusing on different behaviors.

For this reason, in recent years, the term “selector” has been forged to describe a custom filter based on a statistic calculated for the frequency subbands of a signal [16,17]. The third approach is in obtaining a filter by performing a decomposition of the signal in time and frequency [18,19]. Another method is related to the discrete division of the frequency spectrum [20]. The most popular application of this approach is a kurtogram [21,22]; however, over the years, many variations have been proposed, such as the spectral Gini index [23], IFBIα-gram [24], infogram [25], spectral smoothness index [26], and harsogram [27].

There are also other approaches that will produce a custom filter for a given data [28,29]. Such a filter will focus on a certain feature of the data (i.e., impulsiveness) and indicate the frequency bands that contain this feature with a certain proportionality. One can then use this filter to enhance the quality of the signal toward the detection of a given component. There are many already developed selectors, with spectral kurtosis (SK) popularized by Antoni as one of the most famous [30,31,32].

Unfortunately, the selectors are time-invariant; therefore, there are suboptimal solutions in time-varying conditions, especially when one can suspect a changing frequency band occupied by the component of interest. To address this problem, researchers have developed solutions that can react to time-varying conditions, such as adaptive filters [33,34,35,36], and some of those methods can be even implemented in so-called “online forms” so that they can be used in real-time monitoring systems.

In this paper, the author presents an alternative approach that takes advantage of the capabilities of multidimensional analysis. A base for this consideration is the idea of a selector that expands a given scalar statistic, such as kurtosis, to form an object that presents the distribution of this statistic across the frequency domain, thus, allowing the construction of a filter. Here, I applied this concept to generalize the selector even further, adding the aspect of time. Hence, while SK is a kurtosis defined in terms of frequency subbands and by "selecting" the spectrum of the signal acting as a filter, the structure presented in this paper can be described as time-varying spectral kurtosis (TVSK), which is expected to interact with the time-frequency representation of the signal (such as a spectrogram) acting as a multidimensional filter.

The paper is structured as follows: First, the method of simulating the test signal for concept demonstration is discussed in Section 2.1. Then, the spectrogram is defined in Section 2.2 as a base data structure for the analysis. After that, the background of the method is explained in Section 2.3–Section 2.5. Finally, in Section 3, the operation of the method is demonstrated using a simulated signal as well as two real-life signals.

## 2. Methodology

### 2.1. Simulated Signal Construction

The principle of operation of the presented method will be first demonstrated using a simulated signal that is assembled from three components:

- the fault component, which is an amplitude-modulated cyclic impulsive signal,
- a series of frequency-modulated sine waves representing time-invariant frequency components describing the ordinary behavior of machine operation, and
- Gaussian background noise.

A fault component is constructed as a sequence of individual impulses distributed in time with a period corresponding to the modulation frequency $f_{mod}$ of the simulated fault. A single impulse is defined as a decaying harmonic oscillation:

(1) $$g\left(t_{imp}\right)=A\cdot \sin\left(2\pi f_{c}t_{imp}\right)e^{-dt_{imp}},$$

where *A* is the amplitude, $t_{imp}$ is a discrete time lasting only the length of one impulse, $f_{c}$ is the center frequency of the carrier band of the impulse, and *d* is a decay factor for the exponential function. This way, one can define a vector of zeros G(t) of length *l* that will contain properly arranged impulses, where *t* is the full time of the signal. The impulsive component is constructed as follows:

(2) $$G\left(n_{i}:n_{i}+t_{imp}\right)=g,\;where\;n=1:\left(f_{s}/f_{mod}\right):l\;and\;i=1:length\left(n\right)$$

After that, the component is amplitude-modulated to simulate the time-varying operational load that excites the resonant frequency band occupied by the fault component, which, in turn, causes the energy of the impulses to vary. The amplitude modulation is performed as

(3) F=G⊗L,

where *F* is the resulting fault component, *L* is the load profile, and ⊗ denotes the Hadamard product.

The second component of the simulated signal is the set of sines that represent stationary frequencies describing the ordinary behavior of the machine operation, which would be the case for a constant external load. However, in this case, one needs to consider the time-varying load. Hence, the sines were frequency-modulated with the same load profile *L* that is used to amplitude-modulate the fault-related impulses.

The final component of the simulated signal is the background Gaussian noise. The energy was set in a way where fault-related impulses were only partially visible in the signal, and they were also not too clearly visible on the spectrogram, and thus that the case would not be too easy for the analysis.

### 2.2. Short-Time Fourier Transform

I used Short-Time Fourier transform (STFT) as a starting point for the analysis. This is one of the most useful and transparent time-frequency decomposition methods. In the discrete form, this transform is defined as [37]:

(4) $$STFT\left(t,f\right)=\sum_{k=1}^{n}x\left[k\right]w\left(t-k\right)e^{\frac{2j\pi fk}{n}},$$

where w(t−k) is the shifting window and $x_{k}$ is the discrete signal (k=1,2,...,n). Based on that, the spectrogram is defined as:

(5) $$spec\left(t,f\right)={\left|STFT\left(t,f\right)\right|}^{2}.$$

### 2.3. Time-Varying Spectral Kurtosis

The basis for TVSK is an ordinary kurtosis that, for a given signal *X* of length *n*, is defined below:

(6) $$Kurt\left(X\right)=\frac{m_{4}}{{m_{2}}^{2}}=\frac{\frac{1}{n}\sum_{i=1}^{n}{\left(x_{i}-\bar{x}\right)}^{4}}{{\left(\frac{1}{n}\sum_{i=1}^{n}{\left(x_{i}-\bar{x}\right)}^{2}\right)}^{2}}$$

where $m_{4}$ is the fourth central moment and $m_{2}$ is the second sample moment (sample variance). A well-known extension of this statistic is the spectral kurtosis popularized by Antoni [31]:

(7) SK(f)=Kurt(spec(T,f)),

where *T* denotes the full duration of time *t*, and *f* is a narrow range of frequencies contained in a single frequency bin of the spectrogram. In this way, it is possible to determine which frequency bands carry the most impulsive information content. TVSK extends this approach by adding the dimension of time variability to the SK and is defined as

(8) $$TVSK\left(t_{s},f\right)=Kurt\left(spec\left(t_{s},f\right)\right),$$

where $spec(t_{s},f)$ is a segment of a single spectrogram-originated subsignal describing a single frequency bin *f* over a time segment $t_{s}$. In a similar way to the construction of STFT, while the STFT window *t* denotes a series of consecutive samples of an input signal, the length of a window $t_{s}$ for TVSK is a collection of points in the time domain of the spectrogram. In this way, the window and overlap of the TVSK in the time dimension are the input parameters that yield the same function as the window and overlap of the STFT.

Additionally, for $t_{s}=T$ (the TVSK window length set to the full length of the spectrogram in the time dimension), TVSK simplifies to the classic SK. This is why TVSK is a generalization of SK, or, when described from the opposite point of view, SK is a special case of TVSK.

### 2.4. Postprocessing: Median Filter and Resampling

As kurtosis is very sensitive to differences in the input data, it often occurs that two neighboring values of TVSK differ significantly, which, in practice, results in a high local variability of the map. Hence, I apply a two-dimensional median filter to the TVSK to smooth out those highly nonuniform areas. A median filter was selected because it is common that this local variability is induced by a single value diverging from the local trend, resulting in a similar effect to the one known in the field of image processing as salt and pepper noise.

The median filter is a perfect tool to remove those non-uniformities, and testing showed that it performed better than simpler convolution-based filters, such as moving average or Gaussian kernel filters. In practical implementation, the MATLAB${}^{®}$ built-in function medfilt2 was used.

After dealing with local variability, TVSK needs to be resampled in the time domain, as, due to windowing, it has a lower number of time points than the spectrogram. This was achieved using upsampling with Modified Akima piecewise cubic Hermite interpolation [38,39,40]. This method presents several advantages in comparison to other methods:

- It produces undulations that find a balanced middle ground between ‘spline’ and ‘pchip’ (Piecewise Cubic Hermite Interpolating Polynomial) techniques.
- It increases the robustness of Akima’s formula in the edge case of equal side slopes.
- It eliminates a special type of overshoot occurring when the data is constant for more than two consecutive nodes, which is relevant after using a median filter that has a “piecewise-flattening” effect.

I would like to encourage the reader to experiment with different interpolation methods for particular datasets; however, the differences in results are minimal.

### 2.5. Binarization and Filtration

The main idea assumes applying a binary mask to the spectrogram to filter it multidimensionally in the time-frequency domain. In this case, the mask is obtained by performing binarization of the TVSK map using a cutoff threshold for kurtosis values. This is obtained automatically by calculating the kernel density estimate for all values of TVSK and then finding the first local minimum higher than the main distribution mode, which describes the background noise. In such a case, it is assumed that the first distribution mode will be the strongest one, because the impulsive content is not expected to occupy as much area on the time-frequency representation as the background content.

The distribution density is obtained using the kernel density estimator, which is the estimated empirical probability density function of a random variable [41,42]. For real values of the data *x*, the estimated distribution is given by:

(9) $${\hat{f}}_{h}\left(x\right)=\frac{1}{nh}\sum_{i=1}^{n}K\left(\frac{x-x_{i}}{h}\right),$$

where $x_{1},x_{2},\cdots ,x_{n}$ are samples of unknown data, K(·) is the kernel smoothing function, *n* is the sample size, and *h* is the bandwidth. For this example, a Gaussian kernel is used.

The value of the bandwidth is obtained using the so-called *Silverman’s rule of thumb* [42]. For the Gaussian kernel, and the assumption of a Gaussian mixture, the optimal choice for *h* (that is, the bandwidth that minimizes the mean integrated squared error) is

(10) $$h={\left(\frac{4{\hat{\sigma }}^{5}}{3n}\right)}^{\frac{1}{5}}\approx 1.06\hat{\sigma }n^{-1/5},$$

where $\hat{\sigma }$ is the estimator of a standard deviation of the samples and *n* is the number of samples.

When the threshold is found, the areas of TVSK taking lower values are set to 0 (in practice to machine the epsilon value to avoid numerical problems in the following steps), and the areas of higher values are set to 1. After this, the masking of the spectrogram (element-wise multiplication with binarized TVSK, also known as the Hadamard product) is performed as a method of multidimensional filtration. As a result, a new spectrogram is obtained, which is then processed with the Griffin–Lim algorithm that allows us to convert the magnitude spectrogram to a time domain signal [43,44].

## 3. Results

The proposed method is tested using a simulated signal as well as two different real-life signals. The simulated signal is prepared in such a way as to emphasize the conditions of the time-varying load by applying amplitude modulation to the cyclic impulsive component related to local damage (in this way, both the amplitude and bandwidth of the individual impulses change in time) as well as by inserting frequency-modulated sine waves to visualize the effect of reverse-proportionality between the load and rotation speed.

### 3.1. Simulated Signal Analysis

**Figure 1:**
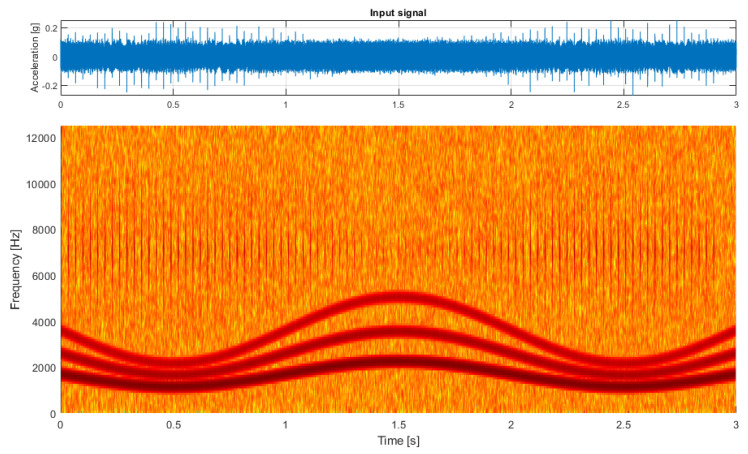
The simulated input signal.

For the first test case, I prepared a simulated signal. This signal describes local damage represented by a cyclic impulsive component in the higher frequency band, a white Gaussian noise with enough energy to cover the impulses for the most part, and a set of three sine components that represent the characteristic frequencies of a given hypothetical machine (see Figure 1, top panel).

The carrier frequency of sine components as well as energy of the cyclic component were modulated to simulate the time-varying loads occurring during the operation of the machine so that lower characteristic frequencies and higher energy of impulses represented the state of higher load. This effect was purposefully exaggerated to emphasize the difference between the low-load and high-load states, which is clearly visible in the spectrogram (see Figure 1, bottom panel). The spectrogram was calculated with a window length of 128 samples, an overlap of 100 samples, and an FFT length of 256 samples.

**Figure 2:**
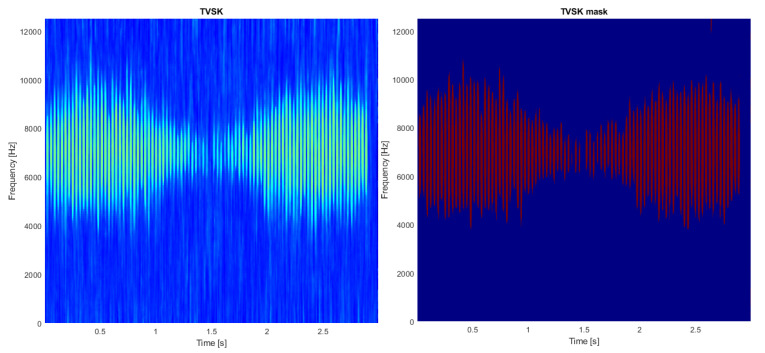
Time-varying spectral kurtosis (**left**) and its resampled version (**right**).

**Figure 3:**
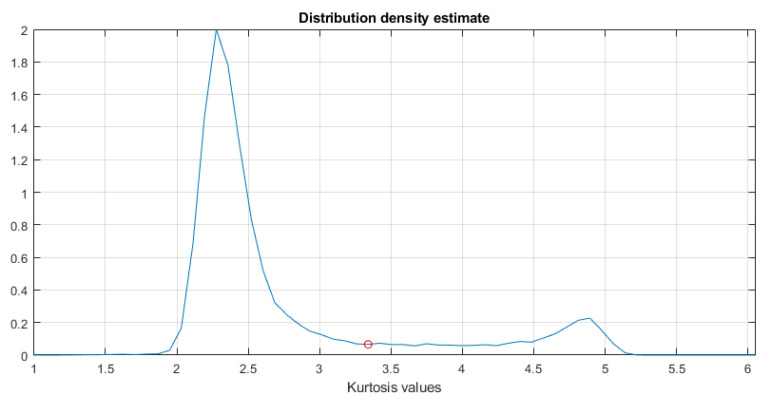
The distribution density estimate with the marked cutoff threshold equal to 3.31.

In the first step, the TVSK is calculated for the spectrogram (see Figure 2, left panel) with a window length of 15 samples and an overlap of 14 samples (both values operate for the time-domain resolution of the spectrogram). Setting a relatively short value of the window $t_{s}$ allows one to distinguish individual impulses, which is the desired effect in this case. Then, the distribution density of the TVSK map is estimated, which allows for discovery of the threshold appropriate to threshold the map (see Figure 3). The result of such binarization is presented in Figure 2 in the right panel.

**Figure 4:**
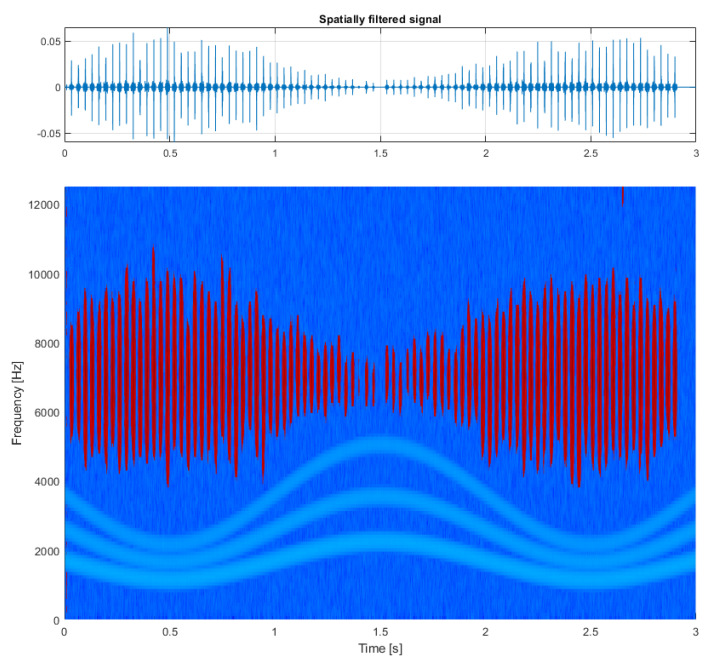
Results of the procedure: multidimensionally filtered signal (**top**) and its time-frequency representation (**bottom**).

**Figure 5:**
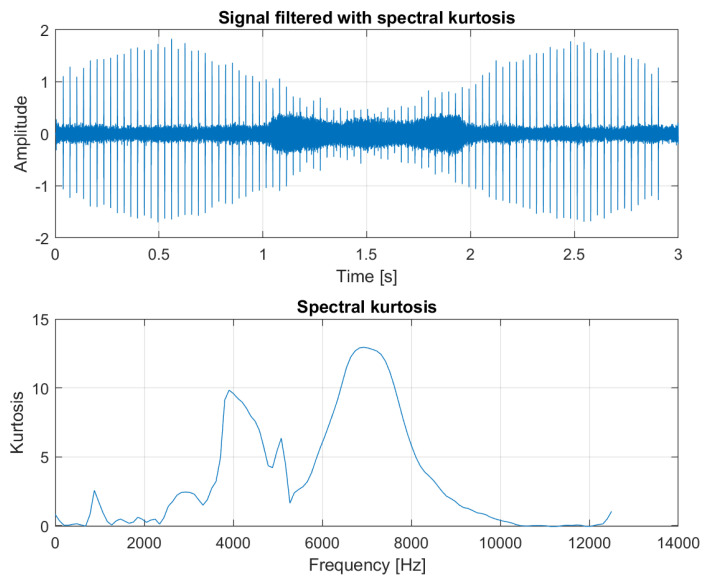
Signal filtered with ordinary spectral kurtosis (**top**) and a plot of spectral kurtosis calculated for the input signal (**bottom**).

In the next step, the spectrogram matrix is multiplied elementwise with the obtained mask, and finally the resulting structure (presented in Figure 4, bottom panel) is fed to the Griffin–Lim phase estimation algorithm, which allows to reconstruct the time series of the obtained result (see Figure 4, top panel). As a comparison, the result of filtering this signal using classical spectral kurtosis is presented in the top panel of Figure 5, with the shape of SK itself on the bottom panel. The difference between the signal-to-noise ratio (SNR) is clearly visible with the value equal to −17.5dBc (decibels relative to the carrier) for the result of multidimensional filtration and SNR=−9.4dBc for the result of filtration with SK.

An interesting effect occurred in Figure 5, where, between 1 and 2 s, the background noise amplitude increased. This comes from the fact that the frequency band in the SK plot that detects impulses overlaps partially with the frequency band that carries the modulated sines. In this segment, the last harmonic frequency is modulated up to 5 kHz, which is partially included within the filter range and remains in the signal after the process of filtration. This is a direct example of why time-invariant filters should not be used for signals recorded in time-varying conditions.

As this is a synthetic signal, I present two additional cases where this method is tested using real-life signals measured on heavy industrial machines.

### 3.2. Real-Life Signal Analysis

**Figure 6:**
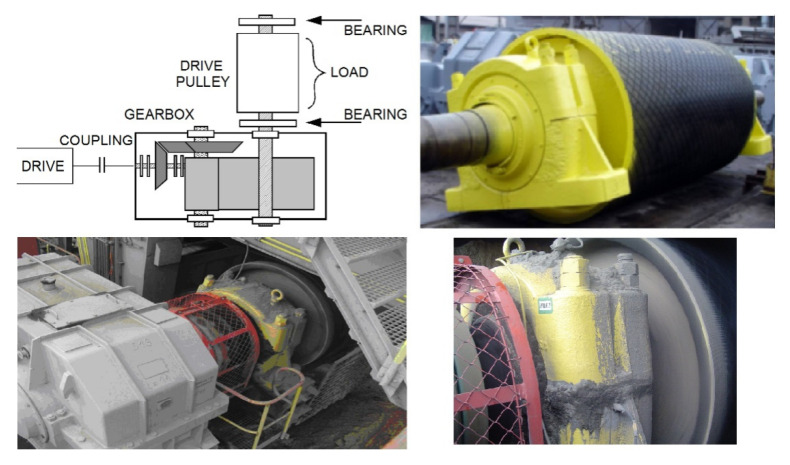
Measurement performed on the bearing in the mine. **Top left**: logical schematic of the belt conveyor drive unit; **top right**: drive pulley with the bearings attached; **bottom left**: drive pulley operating in the system; **bottom right**: vibration sensor mounted horizontally on the right side of the bearing casing.

**Figure 7:**
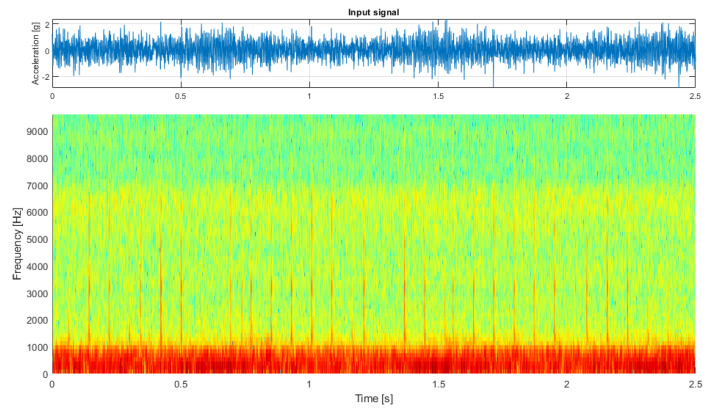
The input signal measured on the drive pulley bearing.

The second test case is the analysis of a real-life vibration signal measured on a rolling element bearing of the drive pulley operating in a belt conveyor driving station (see Figure 6 and Figure 7) with the sampling frequency $f_{s}=19,200$ Hz. It contains a wideband impulsive behavior occupying the frequency band between about 1 and 6 kHz. This component is characterized with the frequency of impulse occurrence equal to 12.69 Hz and represents the local damage of the outer race of the bearing.

Additional amplitude modulation visible in the time series can be observed to occupy the lowest frequency bands, is visible in the spectrogram, and describes the alignment issue of the shaft that is carried by this bearing. For this signal, the spectrogram was calculated with a window length of 64 samples, an overlap of 40 samples, and an FFT length of 128 samples (see Figure 7, bottom panel).

**Figure 8:**
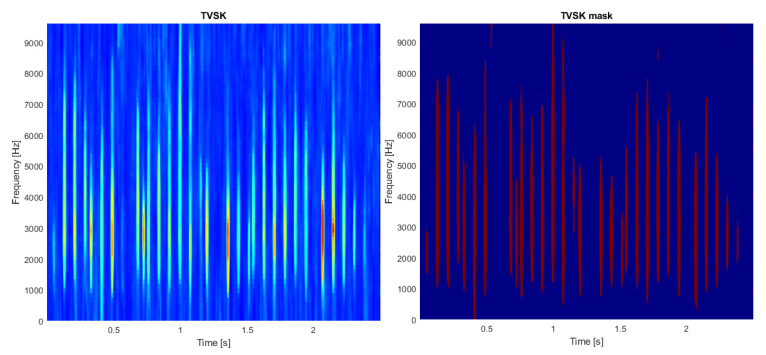
Time-varying spectral kurtosis (**left**) and its mask version binarized with the distribution-derived threshold (**right**, see Figure 9).

**Figure 9:**
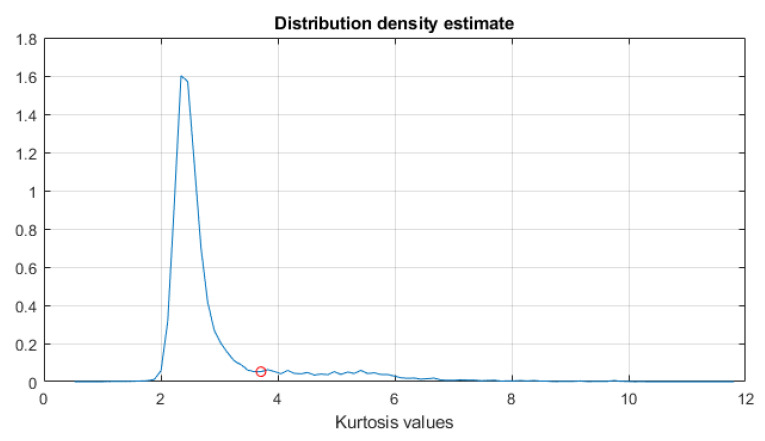
Distribution density estimate with a marked cutoff threshold equal to 3.71.

First, TVSK is calculated with the window length of 16 samples and an overlap of 15 samples. Similarly to the previous case, setting a short TVSK window allowed us to detect and separate the individual impulses (see Figure 8, left panel). In the next step, TVSK was binarized using the threshold obtained from the distribution density estimation (see Figure 9). As a result, the mask functioning as a two-dimensional filter was obtained (see Figure 8, right panel).

**Figure 10:**
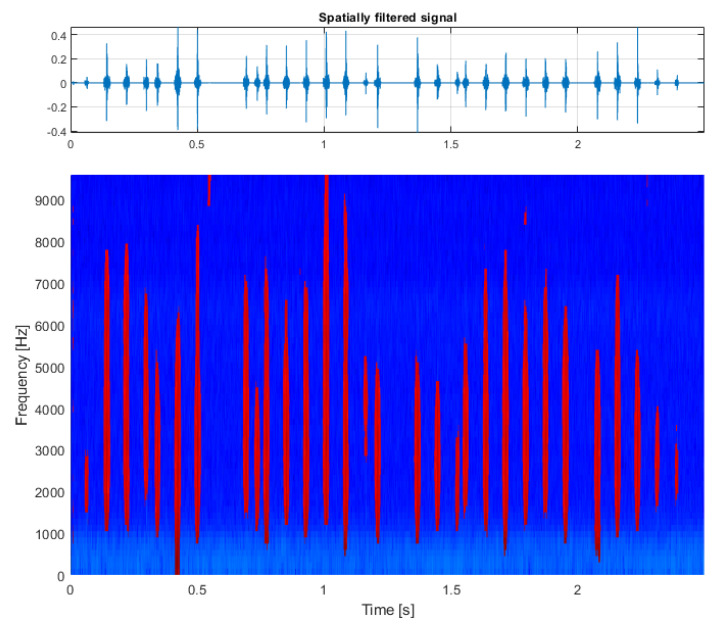
Results of the procedure: multidimensionally filtered signal (**top**) and its time-frequency representation (**bottom**).

**Figure 11:**
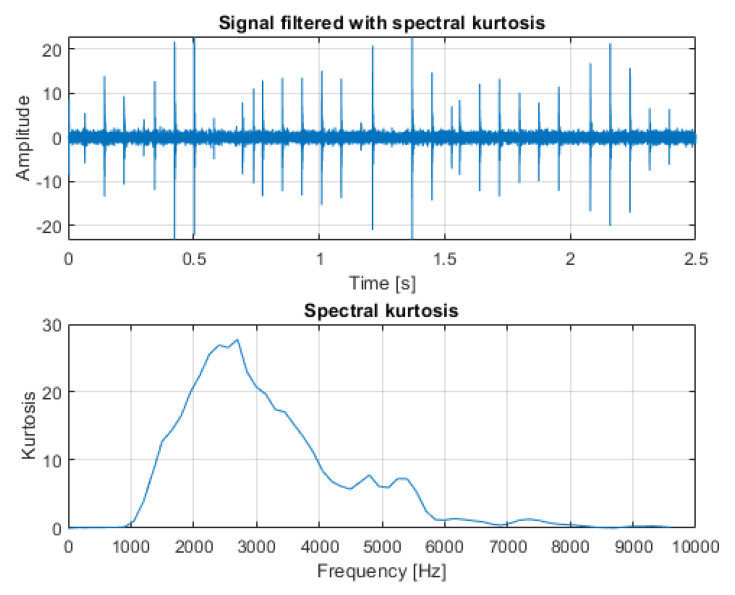
Signal filtered with ordinary spectral kurtosis (**top**) and the plot of spectral kurtosis calculated for the input signal (**bottom**).

The result of filtration is presented on the bottom panel of Figure 10 with the time series reconstructed after estimating the phase of the spectrogram on the top panel. As a comparison, the same signal was filtered with classical spectral kurtosis (see Figure 11, top panel), with the shape of SK presented in the bottom panel. In this case, the differences between the results obtained by the SK and TVSK filtration method were not that large with $SNR_{TVSK}=-20.5\;dBc$ and $SNR_{SK}=-18.2\;dBc$. However, the TVSK method was superior in terms of the effect of the total noise removal in between the individual impulses, which can be beneficial for further analysis.

**Figure 12:**
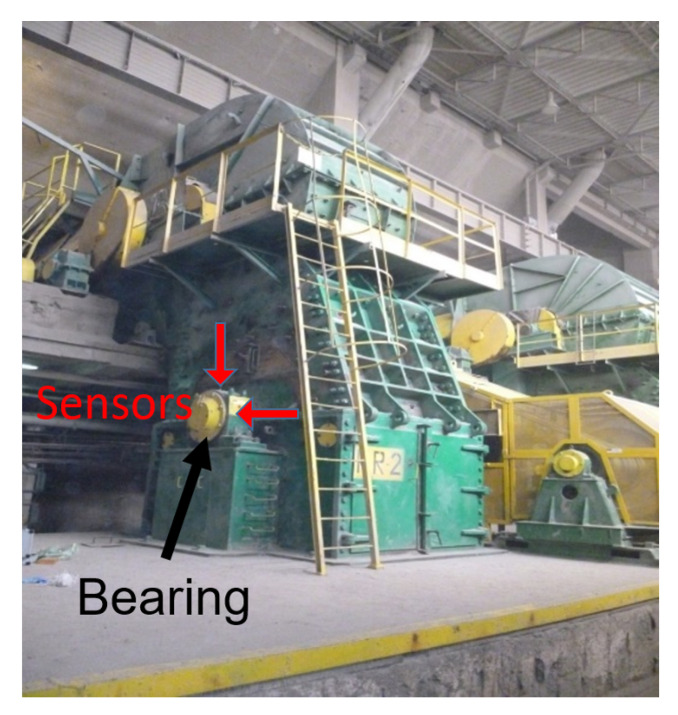
Measurement performed on a real-life crusher during operation. For the purposes of this paper, the signal measured by the vertical accelerometer was used.

**Figure 13:**
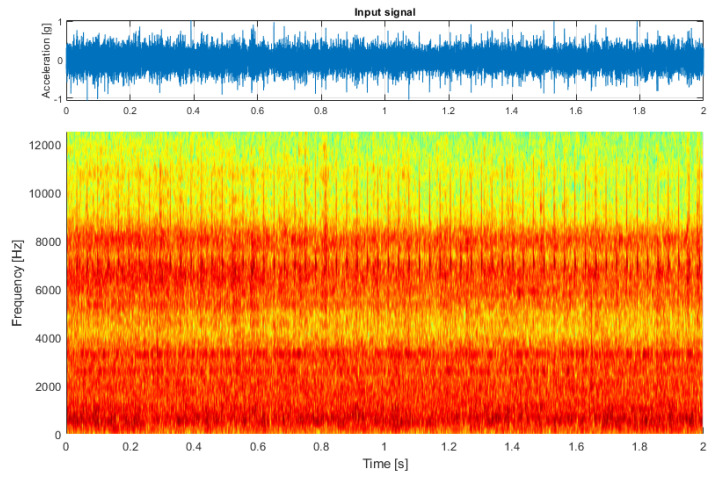
Input signal measured on the crusher bearing.

The third and final case describes the analysis of a vibration signal measured on the bearing of a hammer crusher (see Figure 12 and Figure 13, top panel) with the sampling frequency $f_{s}=25,000$ Hz. This machine processes copper ore by crushing the oversized ore pieces that are too large for the next stage of the process, which is grinding in mills. In this case, the load fluctuation is much more rapid than in previous cases. The load increases when an oversized piece falls in the hammering area and imposes short-time restrictions (just before it is broken into pieces) on the otherwise constant rotational movement of the shaft with attached hammers.

This effect can be observed on the spectrogram (see Figure 13, bottom panel) at about the 0.8 s mark when an oversized piece has been crushed, or just prior to that, when two smaller pieces have been crushed at about the 0.6 s mark. Those effects appear on the spectrogram to be similar to strong wideband impulses, which is partially true, because there are two effects occurring at almost the same time: the ore piece imposes a short movement restriction and is crushed immediately after that, which also translates into an impulse-like signature in the vibration signal.

However, these are not simply the typical high-energy impulses, which is supported by the fact that they are not visible in the time series peaking above the noise, and the normal impulses of similar signatures on the spectrogram should be very strong amplitude-wise in the signal. For this signal, the spectrogram was calculated with a window length of 128 samples, an overlap of 110 samples, and an FFT length of 512 samples.

**Figure 14:**
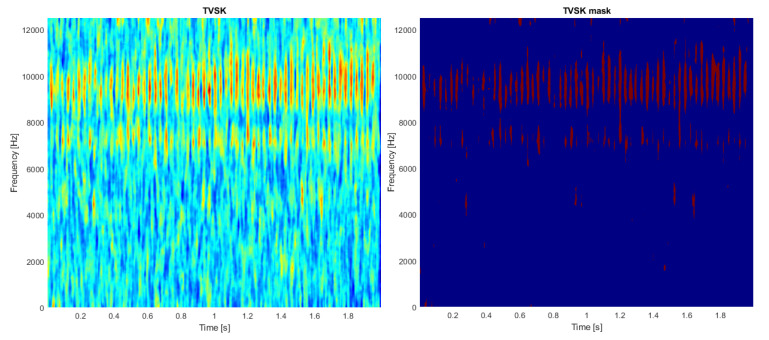
Time-varying spectral kurtosis (**left**) and its resampled version (**right**).

**Figure 15:**
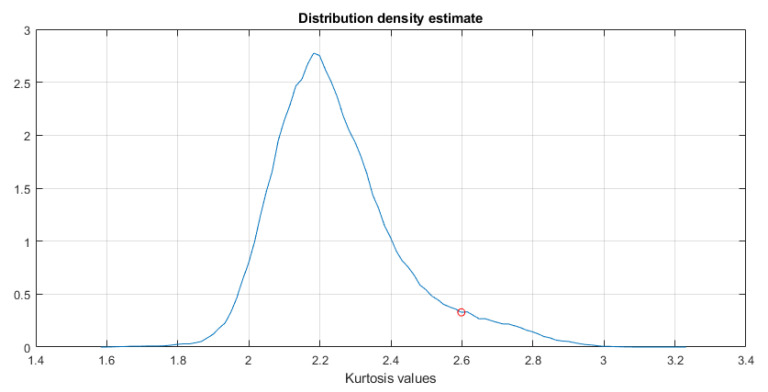
The distribution density estimate with a marked cutoff threshold equal to 2.59.

TVSK has been calculated based on this spectrogram with a window length of 14 samples and an overlap of 12 samples (see Figure 14, left panel). Based on the threshold derived from the distribution density estimation (see Figure 15), a binarized TVSK is presented in the right panel of Figure 14.

**Figure 16:**
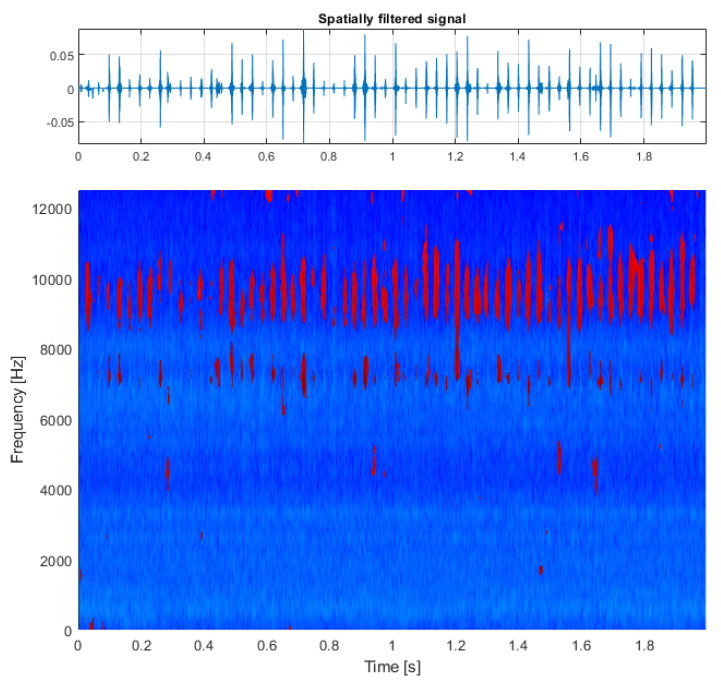
Results of the procedure: multidimensionally filtered signal (**top**) and its time-frequency representation (**bottom**).

**Figure 17:**
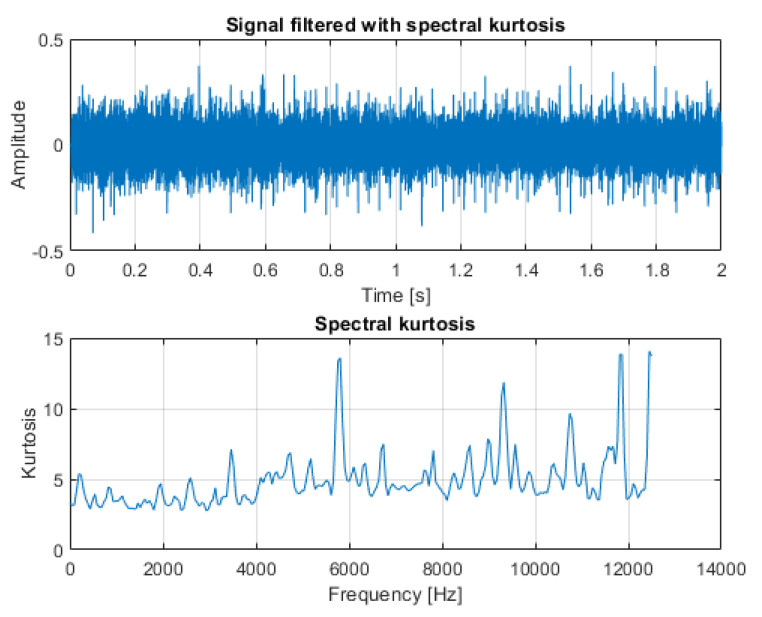
Signal filtered with ordinary spectral kurtosis (**top**) and plot of spectral kurtosis calculated for the input signal (**bottom**).

The outcome of filtration is presented in the bottom panel of Figure 16, and the reconstructed time series on the top panel. For comparison, the result of filtration with the classical SK is presented in Figure 17. In this case, SK was unable to deal with this signal, as one can see the filtered signal is almost the same as the input signal. The profile of SK shows that, while some frequency bands have been noticed, they are too narrow and the overall SK is simply failing, with $SNR_{TVSK}=-17.7\;dBc$ and $SNR_{SK}=-6.2\;dBc$. Again, TVSK succeeded in removing the background content from between the impulses.

## 4. Discussion

The sole idea of calculating the SK over time is not new. Researchers have already attempted this as a base for analysis; however, the approach and purpose were completely different and were not related to multidimensional filtration. Gelman et al. proposed to use this approach to statistically assess the presence of constant carrier frequencies that are not changing over time and consistently raise the kurtosis value, so that the frequencies detected this way can be cross-referenced with the frequencies of rotation that are typical for particular machine elements [45]. In the context of the method proposed in this paper, this effect can be easily achieved by setting a significantly longer time window for TVSK, which will become apparent later in the article. Additionally, in both mentioned papers time-synchronous averaging is required, which helps in detecting constant frequencies.

A similar method was proposed in [46], where the authors tracked the frequency band maximizing SK over time for the purpose of applying a time-varying bandpass filter. Although, in this paper, the SK-related features appear to be changing over time, this again leads to establishing a single bandpass filter for the actual filtering of an input signal. Examples presented in that paper showed that the method left a great deal of noise in the signal and did not clean the signal in between the impulses. Additionally, it is difficult to assess the effect of this method, because the authors presented classic Fourier spectra of the input signals and envelope spectra of the output signals, which are not easily comparable by simply observing the images of plots.

In [47], the authors presented a method that uses SK in an adaptive way. Unfortunately, in this method, the aspect of adaptiveness was not considered in the time domain; therefore, the method is time-invariant and is aimed to help establish an informative frequency band in the signal based on the local maxima of SK.

Combet and Gelman presented, in their paper, how SK allows the construction of a Wiener filter that is known to be an optimal denoising filter [48]. However, it is optimal for filtering out a transient from a stationary background; however, in this case, the background is not stationary, and the components are time-varying. The aspect of variance in the time domain allows the TVSK to act as a noise gate, which is observed as the filter zeroes out the noise between the individual impulses. The second effect is the adaptation to each impulse in time and frequency, which allows the extraction of precisely what is required, and the ability to discard all unwanted background. This is why TVSK excels in such conditions in the precision of selection.

In the presence of those and other articles, the author would like to emphasize the capability of the presented approach for denoising the signal and focusing precisely on the impulses themselves. Of course, the tracking frequencies or construction of optimal bandpass filters can be very useful and lead to successful results; however, as one can see in this paper, multidimensional filtration yields great potential, especially in terms of denoising and precision. Additionally, performing the analysis in the time-frequency domain throughout the entire procedure makes this method suitable for analyzing signals containing a high degree of variability over time.

This is particularly visible in the first example using a simulated signal where the time-varying bandwidth of the individual impulses imposes no difficulty for multidimensional filtration but would be a problem for methods attempting to establish a single filter for the entire signal (see Figure 4). Another example of such advantage can be noticed in the second example (the first real-life signal), which supports the argument that individual impulses can occupy highly varying frequency bands, but regardless of that can be properly identified and filtered without including unnecessary noise (see Figure 10).

**Figure 18:**
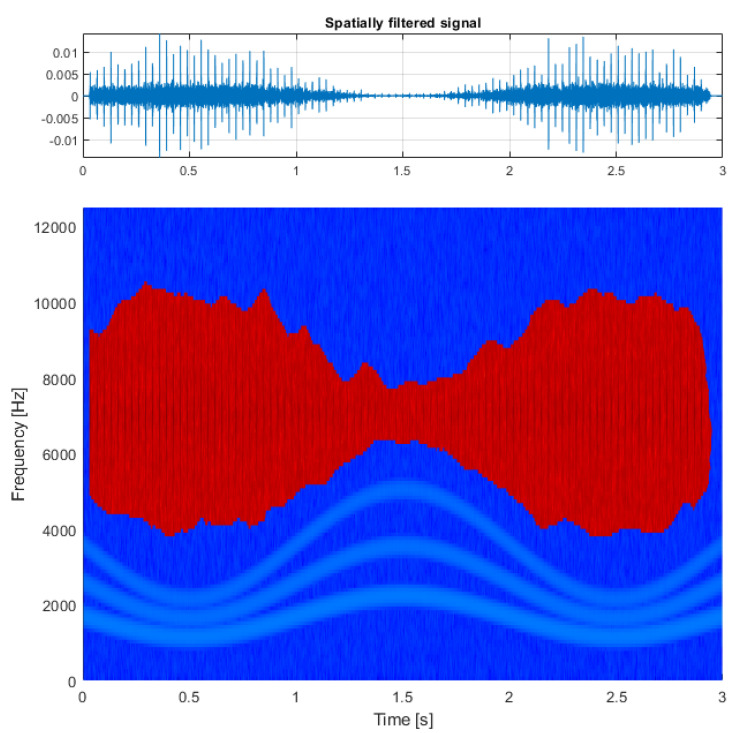
Results of the procedure providing different results when the window and overlap of TVSK are set to higher values.

There is, of course, the possibility to parameterize the TVSK differently. Figure 18 presents the filter shape and the filtration result for window and overlap values set to 50 and 40 samples, respectively. This way, the user has the option to indicate the informative content in a coarser way. Such a result can preserve the entire “section” in a time-frequency sense that can also be useful, i.e., to track the time-varying resonant frequency band, at the cost of retaining the noise content in between the individual impulses.

## 5. Conclusions

In this paper, I present a generalization of spectral kurtosis to a time-varying form called time-varying spectral kurtosis, which provides several advantages. First, it precisely localizes impulses in time, which helps to avoid accumulating an excessive amount of noise when informative events do not occur in the signal (i.e., when there are no fault-related impulses in the signal at a given time). Additionally, it precisely localizes the impulses in the frequency domain, which allows for the avoidance of accumulating noise in the frequency bands that the given impulse does not occupy.

Those effects lead to an increased signal-to-noise ratio in the filtered signal by achieving high selectivity in the time-frequency domain. This approach can be translated to other selectors that are based on statistics other than kurtosis. Future work assumes further study on the postprocessing of TVSK toward a better detection selectivity of individual impulses in highly noisy conditions. Additionally, other multidimensional data representations will be explored in the search of an alternative to the spectrogram in the hopes of achieving even better results.

## Data Availability

Not applicable.
